# How Do Infant Feeding Apps in China Measure Up? A Content Quality Assessment

**DOI:** 10.2196/mhealth.8764

**Published:** 2017-12-06

**Authors:** Jing Zhao, Becky Freeman, Mu Li

**Affiliations:** ^1^ School of Public Health University of Sydney Camperdown, New South Wales Australia; ^2^ China Studies Centre University of Sydney Camperdown, New South Wales Australia

**Keywords:** apps, mobile phone, Chinese

## Abstract

**Background:**

Globally, with the popularization of mobile phones, the number of health-related mobile phone apps has skyrocketed to 259,000 in 2016. In the digital era, people are accessing health information through their fingertips. In China, there are several apps that claim to provide infant feeding and nutrition guidance. However, the quality of information in those apps has not been extensively assessed.

**Objective:**

We aimed to assess the quality of Chinese infant feeding apps using comprehensive quality assessment criteria and to explore Chinese mothers’ perceptions on apps’ quality and usability.

**Methods:**

We searched for free-to-download Chinese infant feeding apps in the iTunes and Android App Stores. We conducted a comprehensive assessment of the accountability, scientific basis, accuracy of information relevant to infant feeding, advertising policy, and functionality and carried out a preliminary screening of infant formula advertisements in the apps. In addition, we also conducted exploratory qualitative research through semistructured interviews with Chinese mothers in Shanghai to elicit their views about the quality of apps.

**Results:**

A total of 4925 apps were screened, and 26 apps that met the selection criteria were evaluated. All 26 apps were developed by commercial entities, and the majority of them were rated poorly. The highest total score was 62.2 (out of approximately 100) and the lowest was 16.7. In the four quality domains assessed, none of them fulfilled all the accountability criteria. Three out of 26 apps provided information covering the three practices from the World Health Organization’s infant feeding recommendations. Only one app described its advertising policy in its terms of usage. The most common app functionality was a built-in social forum (19/26). Provision of a website link was the least common functionality (2/26). A total of 20 out of 26 apps promoted infant formula banner advertisements on their homepages. In addition, 12 apps included both e-commerce stores and featured infant formula advertisements. In total, 21 mothers were interviewed face-to-face. Mothers highly valued immediate access to parenting information and multifunctionality provided by apps. However, concerns regarding incredible information and commercial activities in apps, as well as the desire for information and support offered by health care professionals were expressed.

**Conclusions:**

The findings provide valuable information on Chinese infant feeding apps. The results are concerning, particularly with the relative absence of scientific basis and credibility and the large number of commercial advertisements that are displayed. Apps do seem to be able to provide an opportunity for mothers to access health information and support; it is time for tighter controls on content and advertisements. Ongoing app research and development should focus on implementation of a standard framework, which would drive the development of high-quality apps to support healthy infant feeding through cooperation among academics, health professionals, app users, app developers, and government bodies.

## Introduction

Globally, with the development of new information and communication technologies, mobile phones have reached further than any other communication tool in terms of access [1]. The World Health Organization (WHO) [2] defines mobile health (mHealth) as the use of mobile and wireless technologies to support the achievement of health objectives. The number of health-related mobile phone apps has skyrocketed, reaching 259,000 worldwide in 2016 [3]. In the digital era, people are accessing health information through their fingertips, with health literacy skills improved in all age groups [4]. For example, pregnant women are often disappointed with the quality of their prenatal care and so turn to the Internet and apps to fill the gap [5]. A study in Shanghai, China, found that approximately 40% of 657 surveyed pregnant women chose the Internet as their main source of information on breastfeeding [6]. The number of monthly active users for the most popular parenting app (Babytree) in China reached 8.89 million in 2016 [7]. For new mothers, immediate access to informational resources has been appreciated [8] and also recognized as an essential component of successful maternal role transition [9].

In China, on average, only 28% of infants are exclusively breastfed for 6 months, and the rate of early initiation of breastfeeding, within 1 hour of birth, was only 41% for 2008-2012 [10]. A recent study has shown that of 1350 Chinese infants and young children, aged between 6 and 35 months, only 40% consumed dark green leafy vegetables [11]. Poor food consumption patterns and eating habits in infancy can have an adverse effect on later life, such as a number of chronic health conditions, including being overweight or obese, or having high blood pressure or diabetes [12]. China is now the largest market for infant formula, valued at US $17,783 million, and it is projected to be more than double in value by 2019 [13].

Promoting healthy infant feeding practices is critical for improving nutrition, health, and development of children [14]. The widespread use of apps and the growth in demand for parenting information online, particularly infant feeding information, position apps as a potentially ideal tool to deliver breastfeeding and healthy infant feeding knowledge. However, a content assessment of 46 infant feeding English-language apps from United States, Australia, and United Kingdom revealed poor quality [15]. Notably, studies assessing health-related apps were concentrated in English-speaking countries, whereas countries where apps were downloaded in large numbers such as China, Brazil, and Mexico were relatively neglected [16].

Given the continually increasing number of infant feeding apps and users in China and the lack of existing research to provide detailed app evaluation, exploring the quality of these apps and understanding mothers’ thoughts on them are both essential, particularly in a commercial context of apps being increasingly used for promoting and selling breast milk substitutes [17]. The primary purpose of this study was to perform the quality assessment of free-to-download infant feeding apps available, followed by preliminary qualitative data to accompany with. To take a complete picture of Chinese infant feeding apps, we conducted evaluation on accountability, scientific basis, advertising policy, and functionality of apps; carried out a preliminary screening of infant formula advertisements and e-commerce services within the apps; and collected some preliminary qualitative data on mothers’ opinions on downloaded apps through interview.

## Methods

### App Quality Study

#### App Selection

Parenting apps have been categorized into four catalogues according to their primary feature: *informational* apps are primarily aimed at providing accurate and updated information related to parenting, such as infant feeding, education, and entertainment; *social networking* apps are designed mainly as social platforms to share motherhood experience or images of their infants; *record* apps are used as infants’ growth diary; and *e-commerce* apps are designed as e-stores to sell maternal and baby products [18]. In this study, infant feeding apps are defined as *informational apps* that are primarily aimed at providing information related to infant feeding including breastfeeding and complementary food feeding.

In April 2016, apps were searched from the 360 Mobile Assistant (the premier store for distributing Android mobile phone apps in China) and the Chinese iTunes App Store. Search terms (in Chinese) included infant feeding, baby feeding, breastfeeding, mother + baby, and solid food. Inclusion criteria of apps were as follows: (1) intended for the promotion of healthy infant feeding practices, (2) having stand-alone functionality (ie, not requiring subscription to another program to operate), (3) in simplified Chinese characters or simplified Chinese characters available, (4) updated since 2015, (5) only *informational apps*, (6) targeted at parents of infants and young children, and (7) developed in mainland of China. Exclusion criteria for apps were as follows: not free; in videos, electronic books, audio files, news, and blog forms; designed mainly for social networking and e-commerce or designed as a daily tracker or calculator; and not accessible because of broken or dead links. Each app underwent initial screening based on the description page in iTunes and 360 Mobile Assistant, which consisted of a brief description of the app, user ratings, customer reviewers, and associated screenshot images. Apps that fulfilled the inclusion criteria were downloaded onto an iPhone 6 (for iTunes App Store) and onto a Samsung Galaxy 7 (for 360 Mobile Assistant).

#### Evaluation Scale

On the basis of previous studies and tools used to evaluate the quality of online health information [16,19,20] such as Silberg scale [21], Health On the Net Foundation code (HONcode) principles [22], *Journal of the American Medical Association* benchmarks [21], and DISCERN rating instrument [23], we developed a quality assessment tool (Table 1), including criteria in four domains (accountability, scientific basis, advertising policy, and functionality). Accountability was rated on Silberg’s standards [21]. The 9-point Silberg scale is the most commonly used criterion for evaluating information quality [24]. This includes authorship (ie, the author’s credentials and affiliations), attribution (ie, provision of information sources and references), disclosure (ie, ownership or sponsorship disclosure), and currency (ie, whether the app had been modified in the previous month or last modification date is specified).

Scientific basis domain examined the accuracy of information related to infant feeding regarding the level of adherence to the three main practices from the WHO’s infant feeding recommendations [25]: early initiation of breastfeeding within 1 hour of birth, exclusive breastfeeding for the first 6 months of life, and introduction of nutritionally adequate and safe complementary (solid) foods at 6 months together with continued breastfeeding up to 2 years of age or beyond. Each of the three practices was coded as 0 indicating “incorrect information,” 1 indicating “no information provided,” and 2 indicating “correct information.”

The advertising policy domain was included to determine whether any advertising policy was stated and adopted by the app developer. Advertising policy was assessed against the HONcode, one of the most well-known and widely used quality labels [26]. Each item was coded as 0 indicating “not described the policy” or 1 indicating “described the policy.”

**Table 1:** Quality assessment evaluation criteria.

Evaluation criteria		Maximum score (points)
**Accountability**		9
	Authors credited	1
	Author’s affiliations	1
	Author’s credentials	1
	Information sources	1
	References given	1
	App ownership disclosed	1
	Sponsorship disclosed	1
	App modified in the previous month	1
	Creation or last modification date specified	1
**Scientific basis**		6
	Early initiation of breastfeeding within 1 hour of birth attribution	2
	Exclusive breastfeeding for the first 6 months of life	2
	Introduction of nutritionally adequate and safe complementary (solid) foods at 6 months together with continued breastfeeding up to 2 years of age or beyond	2
**Advertising policy**		4
	Any description on advertising policy that the app developer adopted	1
	Any description on which advertisements are accepted	1
	Any statement that advertisement has to be clearly separated and distinguished from the editorial content	1
	Any statement that promotional information has to be clearly separated and distinguished from the editorial content	1
**Functionality**		8
	Calendar	1
	Baby weight or length record	1
	Graph mensuration of infant growth	1
	Social forum	1
	Internet website links	1
	Reminders to log breastfeeding or bottle feeding	1
	Internal keywords search	1
	Personalized context-based notification and alert	1

**Interview guide.**

Mothers who use an infant feeding app:

- Current breastfeeding and child feeding situation (exclusively breastfeeding, breastfeeding, and complementary feeding)
- Usage of infant feeding app; the reasons to use the app
- Criteria used for choosing infant feeding app
- Attitude toward breastfeeding and infant feeding information in the app
- Preferred content/function in used infant feeding app
- Any influence on mothers’ understanding of breastfeeding and appropriate infant feeding (information/advertisement)
- Mothers’ expectation/issues related to baby feeding, which could be solved by technology (new app function or app function improvement)

Mothers who do not use an infant feeding app:

- Current breastfeeding and child feeding situation (exclusively breastfeeding, breastfeeding, and complementary feeding)
- The main source of breastfeeding and infant feeding information
- The reason why they do not use an infant feeding app

The functionality domain appraised eight app functionality compiled from common functionality criteria used in previous app studies [27,28], such as including a built-in online social forum where women can go to seek infant feeding information and support [29]. We examined the homepage of each app for any commercial banner advertisement and then recorded whether any of these advertisements were for infant formula. Additionally, we screened any e-commerce service in each app and noted any infant formula advertisement.

#### Evaluation Procedure

All apps were downloaded and then coded for each criterion of the four domains, namely, accountability (9 points), scientific basis (6 points), advertising policy (4 points), and functionality (8 points). Each evaluation domain was awarded 25 points equally and 100 points in total for all four domains; each app was then given a weighted score out of 100 points for its fulfillment of the different features of the quality evaluation criteria (Multimedia Appendix 1). One assessor (JZ) conducted all the app quality evaluations using this tool, and the results were verified by the second assessor (ML). Statistical analyses were conducted using SPSS Statistics for Windows, version 22.0 (IBM Corp).

### Interviews

For this exploratory research, we conducted face-to-face interviews to elicit information about mothers’ current use of infant feeding apps and whether these apps had any influence on their understanding of breastfeeding and appropriate infant feeding. In addition, we explored mothers’ current practices and the main obstacles that they faced when using these apps. Ethics approval was obtained from the Human Research Ethics Committee of the University of Sydney, Australia (2016/300).

Semistructured interviews were conducted using an interview guide (Textbox 1) and held with 21 mothers over 14 days in May 2016 in Jiading District Maternal and Child Health Hospital, Shanghai, China. At the waiting area of the child health clinic, women who had given birth in the past year, were over 18 years of age, and competent in Mandarin were invited to take part in the interviews. Interviews were conducted in Mandarin and audiotaped with permission from the women. Interviews lasted for approximately 20 to 30 min each. The four app evaluation domains formed the basis of the interview guide. In addition, questions related to app usability were included. All interviews were transcribed verbatim in full in Chinese and then translated into English. Common concerns and experiences among interviewees in relation to the four app evaluation domains were identified.

## Results

### App Selection

**Figure 1:**
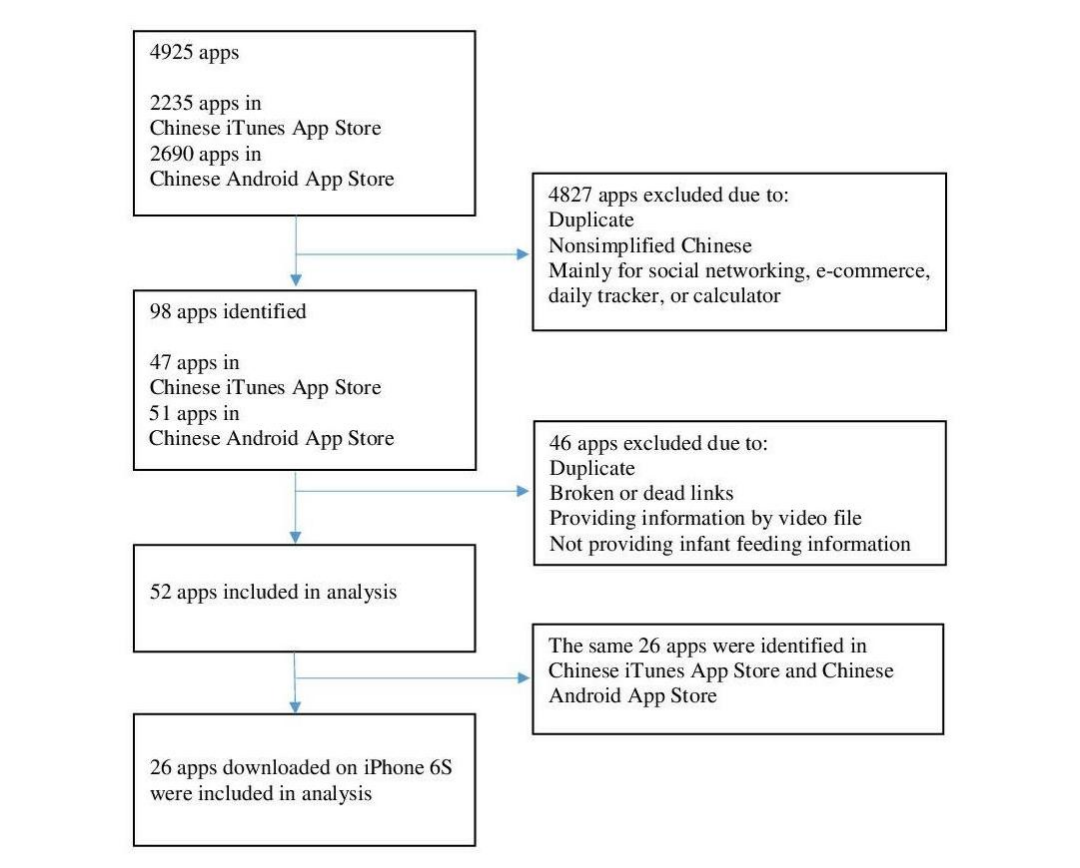
Flow diagram for selection of Chinese infant feeding apps.

The initial search from 360 Mobile Assistant Android App Store (n=2690) and iTunes App Store (n=2235) resulted in a total of 4925 apps (Figure 1). After initial screening based on the inclusion criteria, we deleted duplicate apps (n=152), nonsimplified Chinese apps (n=168), social networking apps (n=829), and e-commerce apps (n=1757) or apps designed for daily record or entertainment (n=1921), and then 98 apps were identified and downloaded to an iPhone 6S and a Samsung Galaxy for initial screening. Apps with the same content under different name, broken or dead links, or apps providing information only by video file were deleted (9 apps from iPhone and 13 apps from Samsung). Following these deletions, we found that the same 26 apps were downloaded in both iPhone and Samsung separately, thus we only used the 26 apps in the iPhone 6S for data abstraction. Furthermore, the final sample included in the quality assessment comprised 26 apps.

### Apps Quality Assessment

#### Sample Characteristics

Of the 26 apps analyzed, all were developed by a commercial entity. Each app was evaluated using the quality assessment tool. Each app’s name in both Chinese Pinyin and English translation, relative ranking, and its original and weighted quality scores for each domain are presented in Multimedia Appendix 1. Overall, mean score (weighted) for the 26 apps was 40.4 (standard deviation [SD] 10.9). *Murumamashouce* ("Mothers’ Handbook of Breastfeeding") received the highest total score of 62.2, followed by *Yuxueyuan* ("Garden of Parenting Knowledge") with a score of 61.9. The lowest scored app was *Baobaochengzhangrili* ("Calendar of Baby’s Growth") with a score of 16.7.

#### Accountability

Of the 26 apps, none fulfilled all the accountability criteria. The mean score for accountability was 3.9 out of 9 (SD 1.4). More than half of the apps were modified within the last month and clearly stated the modification date (16/26, 62%). Approximately 81% (21/26) of the apps only disclosed the app ownership, whereas only 16% (4/25) disclosed app sponsorships. Only one app, *Jiadingmama* ("Adding"), credited the app authors and their affiliations. None of the apps reported authors’ credentials (educational background, professional affiliations, certifications, and past writings).

#### Scientific Basis

The mean score for this domain was 3.4 out of 6 (SD 1.4). Only 3 out of 26 apps provided infant feeding information covering all three practices from WHO’s infant feeding recommendations. Less than half of apps (11/26) provided information on early initiation, whereas the other apps either did not mention early initiation at all or did not include specific timing for initiation. More than one-third of apps (10/26) advised 6 months’ exclusive breastfeeding, and a similar number (9/26) recommended exclusive breastfeeding for the first 4 months. In addition, 7 apps did not specify the time period of exclusive breastfeeding or when solid food introduction should begin. Less than a quarter of apps (6/26) mentioned that the introduction of complementary foods should be at 6 months, together with continued breastfeeding up to 2 years or beyond.

#### Advertising Policy

The mean score for the advertising policy category was 0.9 out of 4 (SD 1.0). Only one app described the advertising policy in its terms of usage; none of the apps identified the advertisement policy accepted or adopted in the app. Approximately 60% (15/26) of the apps did not have a policy clearly separating advertisements from the editorial content. An equal number of apps (15/26) did not have a policy to separate and distinguish commercial promotional information from the editorial content.

#### Functionality

The most popular app functionality was a built-in social forum (19/26), followed by a calendar (16/26). Provision of a website link was the least common functionality (2/26); none of them provided the authoritative infant feeding information website links, such as UNICEF China and WHO China. The app with the most inclusion of functionality was Babytree, which included 7 out of 8 measured items within the functionality domain, whereas *Mengbao* ("Cute Baby"), *Youbaobaola* ("There is a Baby"), and *Baobaochengzhangrili* ("Calendar of Baby’s Growth") only contained one functionality each.

#### Banner Advertisements and E-Commerce Feature

On their homepages, 85% (22/26) apps displayed commercial banner advertisements related to maternal and baby products, such as food and clothing. Four-fifths of these apps (20/22) promoted infant formula advertisements. A total of 12 out of 26 apps included e-commerce, which is an e-store with products directly available for sale. All 12 apps that included e-commerce stores also had infant formula advertisements.

### Preliminary Interview Findings

In total, 21 mothers were interviewed. The mean age of mothers was 31 years (range 25-42 years), and their educational status was predominantly junior college or university level. All respondents were married and currently not working. In the sample, all women owned a mobile phone; the majority of them had used at least one infant feeding information app assessed (16/21); all used infant feeding apps were free to download and were reviewed in app quality study. In addition, 5 mothers did not use infant feeding apps, as they felt that searching questions through the Internet search engine, such as Baidu (a Chinese search engine), is easier than using the app:

I don’t use app, Baidu is very easy to search answers.

Mother 5, age 29 years

Overall, there was a common interest in using apps for accessing infant feeding knowledge. All the mothers (16/21) who use infant feeding apps felt confident using them and had a positive response for using the apps. In addition, mothers noted that they would have no difficulty navigating the apps on their own; they thought it was easy to use infant feeding apps, and nobody mentioned the need for technical support:

These two apps [I am using] both were recommended by a friend, very easy to use.

Mother 18, age 30 years

#### Accountability

In China, the child-raising environment is changing. Unsatisfied with merely providing enough food, which was the major concern of previous generations, mothers now are longing for modern and accurate parenting information:

My mum’s experiences may not be wrong, but they were already outdated.

Mother 2, age 27 years

More than half of the mothers (12/21) revealed that they paid special attention to the source of information and messages to ensure they received the best infant feeding advice. However, some mothers (9/21) were aware of the fact that many apps are developed or supported by commercial companies. A few mothers (5/21) complained about the difficulty of assessing the credibility of information in the apps, as one mother said:

After all, these apps are developed by commercial companies, not a medical institution.

Mother 16, age 30 years

#### Scientific Basis

In addition, some women (5/21) were confused about the information in used apps:

Hm, the parenting information is somehow not reliable, like “4 months” and “6 months to start feeding solid food” both were found in the app.

Mother 1, age 28 years

Mothers (10/21) stated that they could not completely trust the accuracy of apps. This is consistent with the quality assessment that very few apps provided information based on scientific basis. However, because of very limited access to health care providers, which was mothers’ most trustworthy source of infant feeding information, one common precautious measure most mothers took was to combine information from multiple sources, including apps, elders, peers, the Internet, and books. If there was any conflicting information, they would try to make a decision for themselves:

Usually I listen to the suggestions of elders, also communicate with peers and other mothers, and consult with the sellers of maternal products shops, then I conclude those suggestions.

Mother 17, age 26 years

#### Functionality

Mothers (10/21) valued apps that are multifunctional. Features such as reminders and keyword searches were very helpful, which was consistent with apps that scored reasonably in the functionality evaluation. Most of the mothers (13/21) stated that they would use the app frequently because of the helpful functionality, such as reminders, and the social networking features. In addition, the interviews showed that the social forum was an important source of infant feeding information and support. The forum also provided opportunities to help mothers in unusual situations to connect with others with similar experiences, such as when babies are highly allergic or feeding twins. For example, one mother said:

I not only used during pregnancy but also after the baby was born. Because I had twins, quite unusual, I followed closely the women in a similar situation in the discussion room.

Mother 21, age 29 years

However, several mothers (7/21) expressed that information was overwhelming and jumbled in the social forum, and many discussion topics were related to family issues, such as the relationship between a mother-in-law and a daughter-in-law.

Notably, mothers (8/21) described how they used the app to “look stuff up” by internal keyword search functionality, which could provide convenience and immediacy of accessing information right at the time when needed. Some mothers stated that personalized notification was very useful, such as:

...the notification is very helpful, it was set out according to my baby’s height and weight, and is very prompt.

However, there should be a balance, as too many functions in turn could result in poor usability, which was supported by some users’ opinions:

...those apps are too fancy to use.

Mother 1, age 27 years

#### Advertising Policy

Many mothers (8/21) also expressed concern about the commercial advertisements of infant products and the apps. However, some mothers (5/21) noted that they felt powerless over the commercial advertising on account of the fact that the apps were provided by commercial entities. Compared with these types of apps, apps that were supported by government were viewed as more trustworthy:

If it is an official app from government, it can be trusted.

Mother 3, age 29 years

## Discussion

### Principal Findings

In this study, we found a high level of interest and utilization of infant feeding apps, showing the high potential for implementing mHealth in China. The content quality assessment showed that the total score of most apps was low, below 50 points over the four domains. Few apps scored well for measures of accountability, although most of the apps scored full points on currency. This implied that the apps were updated and modified within the month. The quantitative data were supported by interview findings that mothers had a strong willingness for immediacy of information, but many were skeptical about the credibility of content sources. The apps reviewed in this study do not serve as a new tool to support mothers seeking health knowledge, but rather as a new media technology to disseminate formula advertisements and potentially misleading health information.

The content of analyzed apps had low-level adherence to WHO’s breastfeeding and infant feeding recommendations. Additionally, apps largely did not connect users to professional resource links outside of the app. Considering the current large number of app users and strong demand for infant feeding knowledge in China, this should be recognized as a missed opportunity for the promotion of healthy infant feeding practices, particularly exclusive breastfeeding. One reason for the poor scores of scientific basis may be that health professionals and institutions are not involved in app development. All the evaluated apps were developed by commercial companies without academic and government agencies’ input. The development of the apps, therefore, is most likely to be driven by commercial motivation rather than provision of health promotion information. Even though we had deleted all the e-commerce apps (1757/4925, 35.68%) from the initial screening in app stores, we found that almost half (12/26) of the included apps offered e-commerce feature, so parents could still make purchases of products directly through these informational apps. In these e-stores, advertisements of formula often have a clear definition of different stages of the formula, such as “newborn” and “3 to 6 months.” It was reported that in-app advertising has been another source of income among mHealth app publishers [30]. We found that commercial banner advertisements including infant formula were embedded in the homepages of 22 out of 26 apps. Although the Chinese Regulations of the Code were released in 1995 [31], China does not have an operative system to ensure full implementation of the Code [32]. Concern has already been expressed that Internet may be a new source of noncompliance with the Code with respect to infant formula [33]. Given the ubiquity of Internet advertising, the Chinese Government launched Interim Measures for the Administration of Internet Advertising on September 1, 2016, including new regulations on online infant and toddler formula advertising [34], which are expected to be put into practice. As the regulation was launched after this study, we do not have any data relevant to its practical utility in this study.

Another interesting finding is that most reviewed apps (19/26) offered social forum space covering various topics related to mothers and infants. Mothers could not only get information from the social forum but could also build relationships and find support that was not easily offered in a face-to-face setting, particularly in the isolated period after childbirth. However, these social forums are not typically monitored or guided by professionals; we know little about far-reaching health consequences of these forums. Our quantitative finding indicated that the majority of apps had a social forum allowing their registered users to discuss and communicate freely in this space, except for illegal content. This is obviously an essential feature of the current Chinese infant feeding apps. Most interviewed mothers reported that social forum was an irreplaceable source of infant feeding information and support they need. Our finding is supported by other studies that more and more women are seeking peer support and parenting information online, particularly for breastfeeding [35-37]. However, a large amount of information featured on these discussion boards was based on mothers’ experience including infant medical issues, breastfeeding struggles, and the onset of solid food consumption. This information could sometimes be misleading, as they often come from informal sources without scientific basis. In addition, the social forum has limited ability to support health information seeking. One study about social support communication via top online breastfeeding forums in English found that approximately 80% of requests in the discussion board were for information support, but only approximately 60% of them received a message as a result of offering support [29]. From our qualitative study, many mothers said that health workers were the most trusted information source but the least accessed. Therefore, mothers sought reassurance from social forum in apps and other nonprofessional sources, such as their mother-in-law, nanny, or friend. Although mothers appreciated infant feeding information that apps could provide, their views on the information that they got in apps revealed their greater desire to access information and support of health care professionals or government. Indeed, various available but unreliable infant feeding information sources that mothers relied on may help mothers take their own decisions relevant to infant feeding, but it may not be the best choice.

### Future Research

A more in-depth qualitative study is needed to understand how women use feeding apps and how an ideal app could be developed to suit their needs. On the basis of current interviews, participants will be asked to talk aloud while using a parenting app, detailing what type of information they are looking for, where they think they can find it, and what are their reactions to overwhelming information provided through the app, family member, and peer. In-depth, face-to-face and group interviews with probing questions about the app experiences could provide better understanding beyond the four domains. Such a study could inform the development of mHealth interventions in infant health promotion in China. In addition, we plan to conduct a content analysis of breast milk substitute advertising in popular Chinese maternal and baby apps.

### Limitations

This study has several limitations. The study evaluated 26 infant feeding information apps available to the public that satisfied the inclusion criteria. Hence, it is possible that other existing apps could be missed. There is no published comprehensive content quality assessment tool to study health-related apps; therefore, the scope and some of the criteria used in the analysis may impact the variability and comprehensiveness in the scoring.

### Conclusions

This study adds to the understanding of Chinese infant feeding apps in the context of health promotion and online support through mHealth, as well as preparatory investigation of infant formula advertisements on these apps. The result of content analysis and evaluation is not promising, particularly with the relative absence of scientific basis and credibility and the large number of commercial advertisements that are diplayed. Apps do seem to be able to provide an opportunity for mothers to access health information and support, but there is a plea for tighter controls on content and advertisements. In the future, ongoing app research and development should focus on implementation of a standard framework, which would drive the development of high-quality apps to support breastfeeding and healthy infant feeding through cooperation among academicians, health professionals, app users (particularly mothers), app developers, civil society, and government bodies.

## Appendix

Multimedia Appendix 1 Relative ranking and scoring of each evaluated app.

## Abbreviations

mHealth: mobile health
HONcode: Health On the Net Foundation code
SD: standard deviation
WHO: World Health Organization

## References

[1] World Bank (2012). Information and Communications for Development 2012: Maximizing Mobile.

[2] World Health Organization (2016). Monitoring and evaluating digital health interventions: a practical guide to conducting research and assessment.

[3] (2016). Research2guidance.

[4] Powell J, Inglis N, Ronnie J, Large S (2011). The characteristics and motivations of online health information seekers: cross-sectional survey and qualitative interview study. J Med Internet Res.

[5] Kraschnewski JL, Chuang CH, Poole ES, Peyton T, Blubaugh I, Pauli J, Feher A, Reddy M (2014). Paging “Dr. Google”: does technology fill the gap created by the prenatal care visit structure? Qualitative focus group study with pregnant women. J Med Internet Res.

[6] Jiang H, Li M, Wen LM, Hu Q, Yang D, He G, Baur LA, Dibley MJ, Qian X (2014). Effect of short message service on infant feeding practice: findings from a community-based study in Shanghai, China. JAMA Pediatr.

[7] (2016). Bigdata-Research.

[8] Lupton D (2016). The use and value of digital media for information about pregnancy and early motherhood: a focus group study. BMC Pregnancy Childbirth.

[9] Buultjens M, Robinson P, Milgrom J (2012). Online resources for new mothers: opportunities and challenges for perinatal health professionals. J Perinat Educ.

[10] UNICEF (2016). UNICEF Data: Monitoring the Situation of Children and Women.

[11] Yu P, Denney L, Zheng Y, Vinyes-Parés G, Reidy KC, Eldridge AL, Wang P, Zhang Y (2016). Food groups consumed by infants and toddlers in urban areas of China. Food Nutr Res.

[12] Wen LM, Baur LA, Rissel C, Wardle K, Alperstein G, Simpson JM (2007). Early intervention of multiple home visits to prevent childhood obesity in a disadvantaged population: a home-based randomised controlled trial (Healthy Beginnings Trial). BMC Public Health.

[13] Rollins NC, Bhandari N, Hajeebhoy N, Horton S, Lutter CK, Martines JC, Piwoz EG, Richter LM, Victora CG, Lancet Breastfeeding Series Group (2016). Why invest, and what it will take to improve breastfeeding practices?. Lancet.

[14] Black RE, Victora CG, Walker SP, Bhutta ZA, Christian P, de Onis M, Ezzati M, Grantham-McGregor S, Katz J, Martorell R, Uauy R, Maternal and Child Nutrition Study Group (2013). Maternal and child undernutrition and overweight in low-income and middle-income countries. Lancet.

[15] Taki S, Campbell KJ, Russell CG, Elliott R, Laws R, Denney-Wilson E (2015). Infant feeding websites and apps: a systematic assessment of quality and content. Interact J Med Res.

[16] Grundy QH, Wang Z, Bero LA (2016). Challenges in assessing mobile health app quality: a systematic review of prevalent and innovative methods. Am J Prev Med.

[17] Abrahams SW (2012). Milk and social media: online communities and the International Code of Marketing of Breast-milk Substitutes. J Hum Lact.

[18] iResearch (2017). 2017 Report on Population of Maternal & Kid Families in China.

[19] Chen J, Cade JE, Allman-Farinelli M (2015). The most popular smartphone apps for weight loss: a quality assessment. JMIR Mhealth Uhealth.

[20] Jeon E, Park HA, Min YH, Kim HY (2014). Analysis of the information quality of Korean obesity-management smartphone applications. Healthc Inform Res.

[21] Silberg WM, Lundberg GD, Musacchio RA (1997). Assessing, controlling, and assuring the quality of medical information on the Internet: Caveant lector et viewor--Let the reader and viewer beware. J Am Med Assoc.

[22] (2017). Health on the Net Foundation.

[23] Charnock D, Shepperd S, Needham G, Gann R (1999). DISCERN: an instrument for judging the quality of written consumer health information on treatment choices. J Epidemiol Community Health.

[24] Eysenbach G, Lewis D, Eysenbach G, Kukafka R, Stavri PZ, Jimison HB (2005). Design and evaluation of consumer health information web sites. Consumer Health Informatics: Informing Consumers and Improving Health Care.

[25] World Health Organization and UNICEF (2003). Global strategy for infant and young child feeding.

[26] Wilson P (2002). How to find the good and avoid the bad or ugly: a short guide to tools for rating quality of health information on the internet. BMJ.

[27] Li Y, Tan J, Shi B, Duan X, Zhong D, Li X, Qu J (2016). Information and communication technology-powered diabetes self-management systems in China: a study evaluating the features and requirements of apps and patents. JMIR Diabetes.

[28] Abroms LC, Lee Westmaas J, Bontemps-Jones J, Ramani R, Mellerson J (2013). A content analysis of popular smartphone apps for smoking cessation. Am J Prev Med.

[29] Gray J (2013). Feeding on the Web: online social support in the breastfeeding context. Commun Res Rep.

[30] (2016). Research2guidance.

[31] World Health Organization (1981). International Code of Marketing of Breast-Milk Substitutes.

[32] World Health Organization (2013). Country implementation of the International Code of Marketing of Breast-milk Substitutes: status report 2011.

[33] Piwoz EG, Huffman SL (2015). The impact of marketing of breast-milk substitutes on WHO-recommended breastfeeding practices. Food Nutr Bull.

[34] State Administration for Industry & Commerce of the People's Republic of China, 2016 Saic.

[35] Cousineau TM, Rancourt D, Green TC (2006). Web chatter before and after the Women's Health Initiative results: a content analysis of on-line menopause message boards. J Health Commun.

[36] Declercq ER, Sakala C, Corry MP, Applebaum S (2007). Listening to mothers II: report of the second national U.S. survey of women's childbearing experiences. J Perinat Educ.

[37] Dutta MJ, Feng H (2007). Health orientation and disease state as predictors of online health support group use. Health Commun.

