# Supplementary material for: How Do Infant Feeding Apps in China Measure Up? A Content Quality Assessment
Source: JMIR Mhealth Uhealth. 2017 Dec 6;5(12):e186. doi: 10.2196/mhealth.8764 (PMC5738546; doi:10.2196/mhealth.8764)
Supplement: Multimedia Appendix 1 [file mhealth_v5i12e186_app1.pdf]

Relative ranking[a]	App name[b]	Accountability		Scientific basis		Advertising policy		Functionali	
		Original score (9 points)	Weighted score (25 points)	Original score (6points)	Weighted score (25 points)	Original score (4points)	Weighted score (25 points)	Original score (8points)	W (2
1	Muru mama shouce (Mothers' handbook of breastfeeding)	7	19.5	5	20.8	2	12.5	3	
2	Yuxueyuan (Garden of parenting knowledge)	5	13.9	4	16.7	2	12.5	6	
3	Jiading mama (Adding)	6	16.7	5	20.8	0	0	6	
4	Mama bang (Mothers' group)	3	8.3	6	25	2	12.5	3	
5	Xinshou mami (New mother)	2	5.6	6	25	0	0	6	
6	Bangshou mama (Mothers' aid)	3	8.3	4	16.7	2	12.5	3	
7	Mengbao (Cute baby)	3	8.3	5	20.8	2	12.5	1	
8	Yuer ketang (Parenting class)	5	13.9	2	8.3	2	12.5	3	
9	Baobao zhidao (Knowledge abour baby)	6	16.7	4	16.7	0	0	3	
10	Xinshenger huli (New born babies' care)	4	11.1	3	12.5	1	6.3	4	
11	Mami zhidao (Mommy knows)	6	16.7	3	12.5	1	6.3	2	

12	Lmbang（Hot moms' group）	3	8.3	2	8.3	2	12.5	4
13	Baby tree	4	11.1	2	8.3	0	0	7
14	Zhaimama yuer (Raising children at home)	4	11.1	5	20.8	0	0	3
15	Muru yidiantong (Tips for breastfeeding)	3	8.3	6	25	0	0	2
16	Yunyu guanjia (Housekeeper of raising children)	3	8.3	3	12.5	1	6.3	3
17	Youbaobaola (There is a baby)	3	8.3	3	12.5	2	12.5	1
18	Ertong meiri shipu (Menu for children)	3	8.3	2	8.3	2	12.5	2
19	Mengbao fushi (Solid food)	5	13.9	2	8.3	0	0	4
20	Mami yuer (Parenting for mom)	5	13.9	3	12.5	0	0	2
21	Jiankangbaobao ketang (Class of healthy baby)	2	5.6	2	8.3	2	12.5	2
22	Lama yuer (Parenting of hot mothers)	5	13.9	2	8.3	0	0	3
23	Mamawang (Moms' network)	3	8.3	3	12.5	0	0	3
24	Mama shequ (Community of mom)	3	8.3	3	12.5	0	0	2
25	Mami zhangzhongbao (Moms' help)	4	11.1	2	8.3	0	0	2
26	Baobao chengzhang rili (Calendar of baby's growth)	2	5.6	2	8.3	0	0	1
Mean score		3.9	10.9	3.4	14.2	0.9	5.5	3.1

SD		1.4	3.9	1.4	6.0	1.0	6.0	1.6	

[a]**Relative ranking:** ranking was determined according to their total weighted score.
[b]**App name:** Chinese Pinyin (English translation)
